# Perceptions of the Students and Faculty of a Dental College Towards Student Evaluation of Teaching (SET): A Cross-sectional Study

**DOI:** 10.7759/cureus.2390

**Published:** 2018-03-29

**Authors:** Pankaj Gupta, Nikita Bajaj

**Affiliations:** 1 Department of Conservative Dentistry and Endodontics, Nair Hospital Dental College; 2 Dental, Nair Hospital Dental College

**Keywords:** medical education, medical students, teaching faculty, teaching evaluation, medical faculty

## Abstract

Introduction

Student evaluation of teaching (SET) has been in use in some parts of the world for almost a century now. Though India has the highest number of dental colleges in the world, very few colleges employ SET as a tool for improving teaching. The present study was designed to investigate the attitudes of the faculty and students of a dental institute in India, and the differences, if any, that exist between the two major stakeholders.

Materials and methods

Two validated questionnaires for faculty and students about the various aspects of SET were given to consenting participants and the results of the same were statistically analyzed.

Results

Forty-six faculty and 198 students participated in the study. The average age of the students was 21 years while that of the faculty was 37 years. The majority of the faculty thought of SET as a useful educational tool and were open to their teaching being evaluated, though they were divided about SET being used for appraisals. Most students wanted SET to be implemented in their institute and thought that it will improve the teaching being rendered to them.

Discussion

On most aspects, like the when, how often, its mode of administration, and the format of SET, there was an agreement amongst the students and faculty. They differed significantly on the visibility of SET results, where most faculty felt that the results of SET should only be known to the faculty. This can be attributed to apprehensions among the faculty about SET.

Conclusion

The present study concludes that SET is perceived as a useful tool by the students and faculty of the studied institution.

## Introduction

Student evaluation of teaching (SET) has been used as an accountability tool in higher educational institutes from as long back as 1920 in some North American universities [1]. SET has been successfully used as a tool to gauge the satisfaction of students with the education rendered to them [2-5]. It has also been used in career advancement decision-making by the administration of colleges and universities and also may be used as a tool to help students select courses [4,6]. The ultimate aim of dental education is to produce competent dental surgeons and oral physicians, and the quality of education rendered has the greatest influence on this outcome. A sound SET process acts as a quality control tool and a feedback mechanism for the faculty to make changes and improvements in the education system. 

Unfortunately, in India, to date, few dental colleges and universities implement SET as a routine practice, in spite of its proven benefits. This contrasts the situation in the US, where almost 81% of dental schools use it as a routine practice [7]. This is even more disheartening since India has the highest number of dental colleges in the world. The governing body of the dental profession in India, the Dental Council of India, has no provisions for SET to be conducted in various dental colleges and universities.  

The present study was designed to investigate the perceptions of the students and faculty of a dental college in Mumbai, India, on student evaluation of teaching and its various aspects.

## Materials and methods

The study was approved by the institutional ethics committee. The minimum sample size for the study to be valid was calculated, keeping the total strength of the faculty and student body as the entire population, with a confidence interval of 95% and a margin of error of 5%. The minimum sample size thus calculated was 184 for students and 45 for faculty. Two questionnaires were designed, one for the faculty and the other for students; these questionnaires were content and face validated by an expert in the field of medical education, who checked whether they were in line with the objectives of the study. The questionnaires were first piloted among four faculty members and 10 students. The results of the pilot were not included in the final sample. The questionnaires can be found in the appendices. All full-time faculty members and students (both undergraduate and postgraduates), ie., the entire population of the institute, were invited to fill the questionnaire. Those who consented to participate were given a copy of the questionnaire or the web link to the same after signing an informed consent form. A total of 198 students and 46 faculty members returned the filled questionnaires. The faculty questionnaire had 14 items and was divided into three parts. The first part had three questions and inquired about the age, gender, and teaching experience of the respondent. The second part had five questions about their perceptions on SET, and if it should be used for appraisals and promotions. Finally, the third part had six questions that were common to the faculty and student questionnaires, which inquired about how often SET should take place, who should participate in it, what should be the format, how it should be administered, who should have access to the results, and if it should be mandatory for students to participate. The student questionnaire had 11 items and was also divided into three parts. The first part consisted of three items inquiring about the age, gender, and year of study of the respondent. The second part consisted of two items inquiring about the student’s opinion about SET being implemented in their institute and the reasons for the same if the answer was ‘yes’ to the previous question. Third part consisted of the same six questions as in the faculty questionnaire. The paper and pencil responses were entered into Google forms (Google, Mountain View, California, US), and the results were downloaded as an Excel file (Microsoft, Washington DC, US), which were imported to  SPSS vrs21 (Statistical Package for Social Sciences, IBM, Armonk, New York). Descriptive statistics were drawn to throw light on the respondents' characteristics. Mean, standard deviation, and ranges were calculated for the last six questions from both the faculty and student questionnaires about the various attributes of SET. Chi-square test was used to find the differences in the responses of students and faculty.

## Results

Forty-six faculty and 198 students returned the filled questionnaires. The faculty population consisted of 41.3% females and 58.7% males, while the student population consisted of 74.7% females and 25.3% males.

The average age of the faculty was 37 years while the average student age was 21 years. The average teaching experience of the faculty was 9.5 years.

Of the faculty participants, 87% were open to the teaching they provided being evaluated by the students, while 78.3% of the faculty thought that students would like to evaluate the teaching rendered to them. Most faculty (89.1%) were of the opinion that SET will be a useful tool to improve education, and 89% of the faculty were willing to make changes in their teaching methodology based on the feedback received.

The question about linking appraisals and promotions to students feedback met with a variable and divided response as shown in Table 1.

**Table 1:** Linking appraisals and promotions to student evaluation

Should student evaluation of teaching be used for performance appraisals and promotion decisions?		
	N	Percentage
Strongly agree	5	10.9
Agree	19	41.3
Neutral	4	8.7
Disagree	6	13.0
Strongly disagree	12	26.1

Most students (92.9%) wanted SET to be implemented in their institute. Students wanted SET to be implemented for one primary reason, i.e., they felt that the faculty will become aware of what students feel about the quality of education rendered to them and this, in turn, will bring about a change in the same (38.4%) thus improving the quality of teaching.

It was also observed that as the students progressed from the first year to part three of MDS (master's in dental surgery), there was a change in why they thought SET should be introduced. Significantly, more MDS part three students responded with “I feel empowered by filling SET” as compared to the first year, where the predominant response was “Faculty becomes aware of the way students feel about the quality of their teaching, which can bring about a change in how the course will be taught by them." This difference was statistically significant (p=0.0012).

The gender distribution and the results of the six questions about the format, frequency, participation, and method of conducting SET were similar among students and faculty (Table 2).

**Table 2:** Response of students and faculty (percentage)

		Students	Faculty
Gender			
Male		25.3	58.7
Female		74.7	41.3
1. Would you like SET to start in your institute?			
Yes		93.4	87
No		1	6.5
Maybe		5.6	6.5
2. How often should SET take place in your institute?			
After every lab, lecture, and clinic		12.6	23.9
Monthly		33.8	21.7
Fixed intervals		40.9	26.1
Term end		12.6	28.3
3. What should be the format of SET?			
Open-ended questions		37.4	43.5
Closed-ended questions		3.5	8.7
A combination of open and closed-ended questions		48	34.8
A simple scoring		11.1	13
4. Whom do you think should complete the SET?			
All students of the class		83.8	63
Randomly selected half the class		10.6	17.4
Random fixed percentage of the class		5.6	19.6
5. Who should know the SET results?			
Displayed for all to see		19.7	13
To be known to the faculty only		10.1	69.6
To be known to both the faculty and the students		70.2	17.4
6. How should SET be conducted?			
Online		50.5	39.2
Paper and pencil		27.3	30.4
No preference		22.2	30.4
7. Would you want student evaluation of teaching (SET) to be mandatory?			
Yes		64.6	58.7
No		35.4	41.3

## Discussion

The present study was aimed at exploring the attitudes of students and faculty at a dental institute where evaluation of teaching is not in practice.

From the results of the present study, it can be inferred that both the faculty and the students have an overall positive attitude towards implementing SET at the institute. Most faculty (89.1%) responded affirmatively about making changes in their teaching methodology based on SET feedback. Most students also felt that the faculty will make changes in their teaching methodology, thus making the course better.

When asked about how frequently SET should take place, there was a difference of opinion between the faculty and students. The faculty were almost equally divided between all four options from “after every lab or lecture” (23.9%) to “term end” (28.3%). In contrast, students were majorly divided between “monthly” (33.8%) and “fixed interval” (40.9%). 

There was a consensus among students and faculty about the prefered format for SET. Most faculty and students were of the opinion that either an open-ended SET questionnaire or a combination of open and closed-ended questions should be used. This definitely demonstrates a positive view, as it has been shown by a previous study that neither a closed-ended SET method nor a simple scoring scale has a high value when it comes to interpreting the results of SET and making changes accordingly [8].  

On the question of who should fill SET, most faculty (63%) and students (83.8%) felt that the entire class should fill it, though there was a statistically significant difference between the two, with students more strongly feeling that the entire class should engage in the process. This difference can be attributed to the fact that the faculty may perceive the process of SET to be time-consuming, and may thus want the evaluation to be filled by a fixed percentage of the class.

Questions regarding the way SET should be administered met with similar responses from the students and faculty, with the majority of both groups indicating a preference towards online SET administration, though students felt more strongly than the faculty about this. This can probably be attributed to the comfort, convenience, and flexibility that online administration provides to the user, and to which the current generation of students are accustomed. Online administration had the added advantage of saving valuable class time.

Most students (64.6%) and faculty (58.7%) wanted SET to be made mandatory for students. This again reflects the positive attitude of stakeholders towards SET.

**Figure 1:**
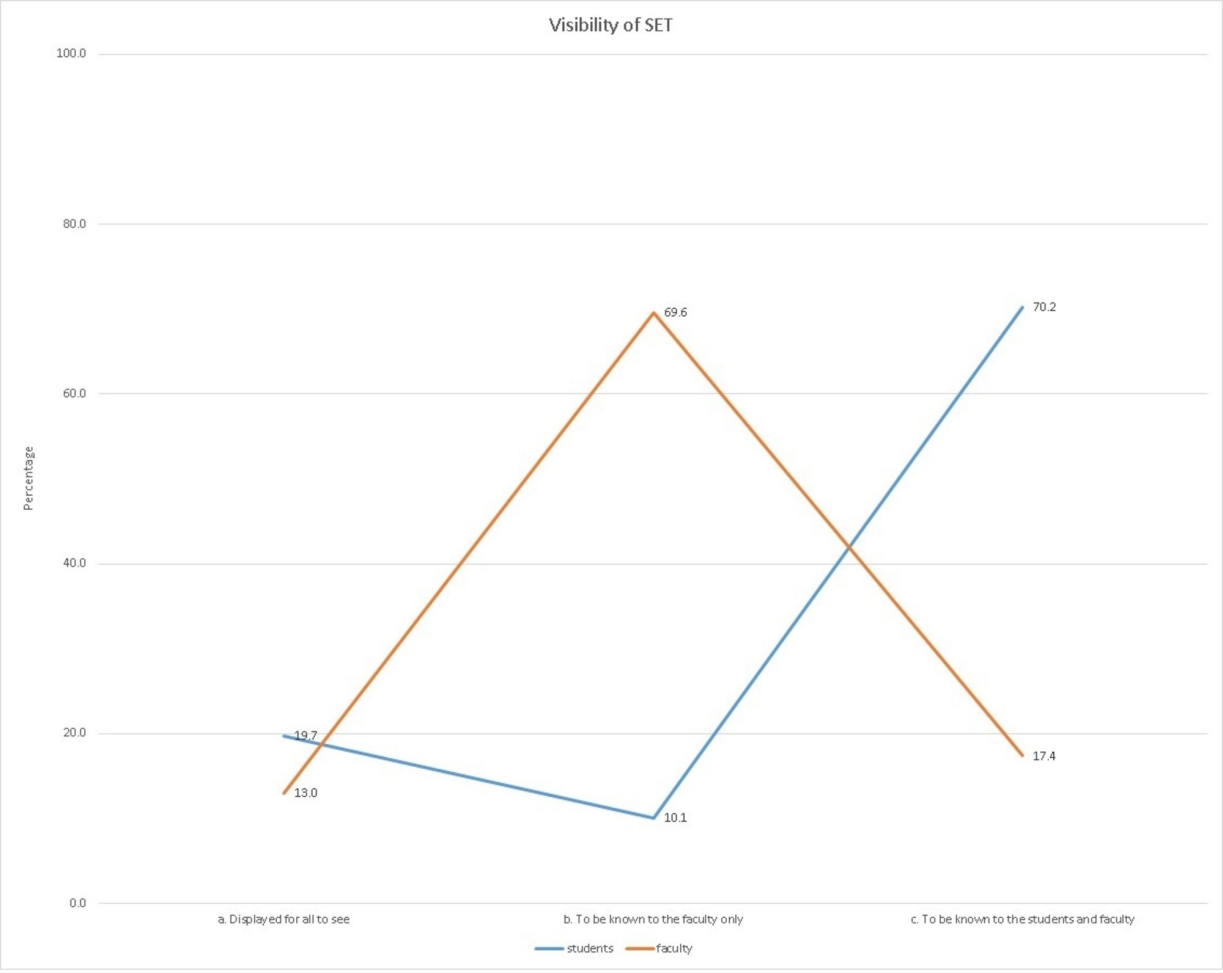
Visibility of results of student evaluation of teaching (SET)

There was an interesting contrast in students' and faculty members' responses when they were asked who should know the results of SET. About 70% of the faculty felt that the results should be known only to the faculty, while around 70% of the students felt that it should be known to both the students and faculty. Less than 15% in both the groups felt that the results should be displayed for all to see. This stark contrast between the perceptions of the faculty and students can be attributed to the fact that since the faculty have never been evaluated before, they may have apprehensions that a negative outcome on SET may tarnish their image as a teacher. This can also possibly be because many faculty members perceive SET to be a personal evaluation of them by students, and not of their teaching. This attitude will probably change once SET becomes a routine part of the teaching curriculum, and the faculty understand that SET is a tool for improving one’s teaching and is not a judgment of one as an individual. The difference in the perceptions can be appreciated in Figure 1.

**Figure 2:**
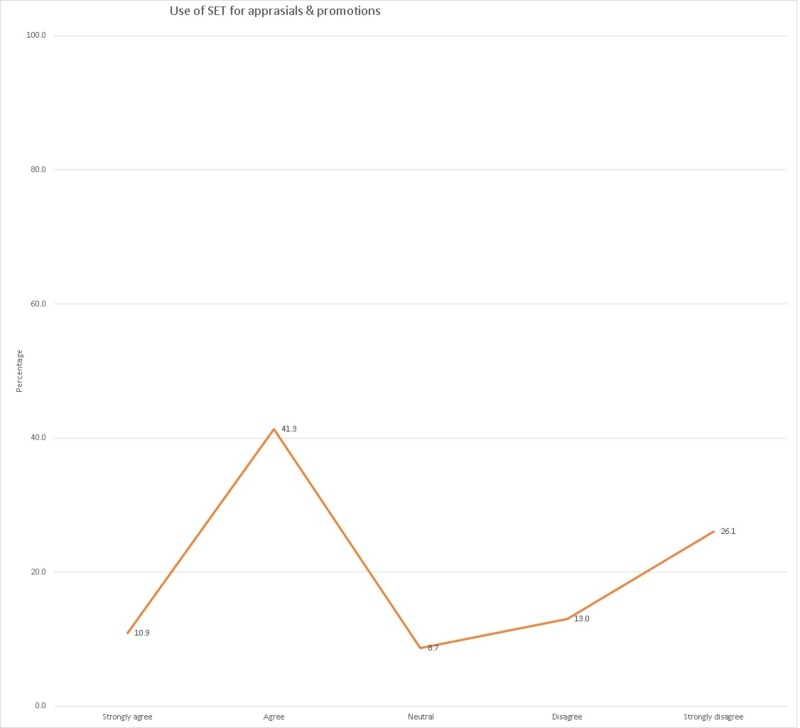
Linking appraisals and promotions to student evaluation

The question about linking appraisals and promotions to SET provoked a divided response amongst the faculty (Table 1), which pointed to probable apprehensions related to the process of SET and its results. Formal training for students and faculty will go a long way in allaying these apprehensions. It is graphically illustrated in Figure 2.

## Conclusions

The overall results of the study indicate that faculty and students regard SET positively, and both stakeholders perceive it as a positive tool for improving the teaching and learning process. 

## Appendices

FACULTY SURVEY

1.      Age

2.      Gender

a.       Male

b.       Female

3.      How many years have you been teaching for?

­­­­___________ years.

4.      In your opinion, do you feel student evaluation of teaching (SET) will be a useful tool for improving education?

a.       Yes

b.      No

5.      Would you like your teaching to be evaluated by students?

a.       Yes

b.      No

6.      Do you think that students will like to evaluate the teaching by the faculty?

a.       Yes

b.       No

7.      Based on SET, will you make changes in your behavior or teaching methodology?

a.       Yes

b.      No

8.      Should student evaluation of teaching be used for performance appraisals and promotion decisions?

a.      Strongly agree

b.      Agree

c.       Neutral

d.      Disagree

e.      Strongly disagree

9.      How frequently should student evaluation of teaching (SET) happen in your institute?

a.       After every lecture, lab and clinic

b.      Monthly

c.       Fixed intervals

d.      Term end

10.      How would you like the format of the student evaluation of teaching (SET) to be?

a.       Open-ended (specific inputs can be given by the students)

b.      Closed-ended (no place for specific inputs, only fixed questions to be answered)

c.       Combination of a and b

d.      A simple scoring

11.      Who do you think should fill the student evaluation of teaching (SET)?

a.       All students of class

b.      Randomly selected half the class

c.       Random fixed percentage of the class

12.      Would you like the results of the student evaluation of teaching (SET) to be

a.       Displayed for all to see

b.      To be known to the faculty the only

c.       To be known to the students and faculty

13.      In your opinion, what should be the mode of student evaluation of teaching (SET)?

a.       Online

b.      Paper and pencil

c.       No preference

14.      Would you want student evaluation of teaching (SET) to be mandatory?

a.       Yes

b.       No

STUDENTS SURVEY 

1.      Age

2.    Gender

a.       Male

b.      Female

3.      Which year of BDS (bachelor's of dental surgery) or MDS (master's of dental surgery) are you in?

a.       I

b.      II

c.       III

d.      IV

e.       Intern

f.        MDS 1

g.      MDS 2

h.      MDS 3.

4.      Would you like student evaluation of teaching (SET) to be started in your institute?

a.      Yes

b.       no

5.      What is the prime reason to start student evaluation of teaching (SET) in your institute?

a.       Change of the teaching methodology

b.      You feel empowered by filling SET

c.       Faculty becomes aware of the way students feel about the quality of their teaching which can bring about a change in how the course will be taught by them

d.      It will improve the course

6.      How frequently should student evaluation of teaching (SET) happen in your institute?

a.       After every lecture, lab and clinic

b.      Monthly

c.       Fixed intervals

d.      Term end

7.      How would you like the format of the student evaluation of teaching (SET) to be?

a.       Open-ended (specific inputs can be given by the students)

b.      Closed-ended (no place for specific inputs, only fixed questions to be answered)

c.       Combination of a and b

d.      A simple scoring

8.      Who do you think should fill the student evaluation of teaching (SET)?

a.       All the students of class

b.      Randomly selected half the class

c.       Random fixed percentage of the class

9.      Would you like the results of the student evaluation of teaching (SET) to be –

a.       Displayed for all to see

b.      To be known to the faculty only

c.       To be known to the students and faculty

10.      In your opinion, what should be the mode of student evaluation of teaching (SET)?

a.       Online

b.      Paper and pencil

c.       No preference

11.      Do you think that student evaluation of teaching (SET) to be mandatory for the students?

a.       Yes

b.       No
